# Is the emergence of the zoonotic Langya virus amidst COVID-19 and monkeypox a cause for concern?

**DOI:** 10.2217/fvl-2022-0175

**Published:** 2023-01-27

**Authors:** Chandan Kumar Thakur, Jog Bahadur Adhikari, Nitin Gupta, Prakash Ghimire, Meghnath Dhimal

**Affiliations:** 1 Nepal Health Research Council, Ramshah Path, Kathmandu, Nepal; 2 Department of Surgery, Patan Academy of Health Sciences, Lagankhel, Lalitpur, Nepal; 3 Department of Infectious Diseases, Kasturba Medical College, Manipal Academy of Higher Education, Manipal, India; 4 Central Department of Microbiology, Tribhuvan University, Kathmandu, Nepal

**Keywords:** COVID-19, *Henipavirus*, Langya virus, monkeypox, paramyxoviridae, viral pneumonia, zoonotic virus

After the two major viral outbreaks of COVID-19 and monkeypox, a new viral outbreak due to the Langya virus (LayV) was identified in febrile patients from the eastern Chinese provinces of Shandong and Henan [1]. LayV, named after the town Langya in Shandong Province, is a phylogenetically distinct zoonotic *Henipavirus* [2]. The *Henipaviruses*, such as Nipah and Hendra, are enveloped, negative-strand RNA viruses belonging to the Paramyxoviridae family [3]. The Hendra virus was responsible for seven cases, primarily in veterinarians dealing with sick horses in Australia [4]. The Nipah virus is becoming increasingly prevalent internationally, with outbreaks commonly recorded in Bangladesh and India. The initial epidemic was recorded in Malaysia and Singapore among humans who had direct contact with pigs [5]. Recent outbreaks, however, are considered to be the result of food being contaminated with the urine or saliva of sick bats [4,6]. Also, cases of human-to-human transmission (including nosocomial transmission) have been described in the Indo–Bangladesh outbreaks. Nipah virus cases were associated with various complications, including encephalitis, acute respiratory distress syndrome and multiorgan dysfunction syndrome leading to high fatality rates, which were significantly higher when compared with COVID-19 and monkeypox [7–10].

According to a recent sentinel surveillance report (April 2018–August 2021) from China, 35 febrile patients, mostly farmers and factory workers with a recent history of animal contact, tested positive for the LayV virus. Most patients were from the Shandong and Henan provinces in eastern China. A distinct phylogenetic LayV was isolated from a 53-year-old febrile patient’s throat swab utilizing a metagenomic approach. The acute and convalescent serum samples from 14 infected individuals demonstrated a fourfold rise in IgG antibody titer. The majority of patients presented with fever and other constitutional symptoms [2]. It is important to note that China is undergoing a huge wave of COVID-19 at present, which can present with similar manifestations [11]. Similar to the Nipah virus, patients with LayV had thrombocytopenia, leukopenia and altered liver/kidney functions. These laboratory findings can be seen in COVID-19 as well, but they are not as common. Similar to Nipah virus disease and COVID-19, pneumonia is an important complication of LayV infection. It was noted that the patients with LayV pneumonia had an increased viral load compared with those who did not have pneumonia.

In a serosurvey of domestic animals, goats and dogs were seropositive at 2 and 5%, respectively. Out of the 25 species of wild small mammals that were examined, LayV RNA was primarily detected in shrews (27%) [2]. This is similar to fruit bats being identified as the primary reservoir in Nipah outbreaks in the Indian subcontinent. The LayV genomic study revealed that the virus genome consists of 18,402 nucleotides and is closely related to the *Mojiang henipavirus*. The Mojiang virus is rat borne and was responsible for three deaths in Chinese miners in 2012 due to pneumonia [12]. The reported cases may be only the tip of the iceberg. Extensive contact tracing of some of the diagnosed cases did not reveal any indication of human-to-human transmission. Also, no aggregation of human cases were noticed. However, these numbers are small, and it is difficult to completely rule out current or future human-to-human transmission possibilities [13]. As we witnessed with COVID-19 and monkeypox, viruses are capable of rapid mutation, and human behaviours frequently affect the course of viral epidemics. It is, therefore, vital to focus on this epidemic and prevent the virus from spreading. Other than some reports of the activity of ribavirin and favipiravir on the Nipah virus, there are no approved drugs or vaccines for *Henipaviruses* [14]. There are no effective treatments for LayV. However, unlike Nipah and Hendra viruses, no fatality has yet been reported for LayV. There have been no effective vaccines for any of the *Henipaviruses* that can be used for LayV. The only sensible public health interventions at present are surveillance (human and animal) and restricting contact with affected animals.

Taiwan’s health department is currently monitoring the spread of LayV near the China–Taiwan border by instituting standardized molecular assay-based diagnostics [15]. Nepal also shares a northern border with Tibet and China and has a trade relationship with the country. Agriculture and farming are significant sources of income and the economic backbone of Nepal, employing more than 66% of the population and accounting for a third of the GDP [16]. Farmers are considerably closer to their livestock, which is a vital source of sustenance. Furthermore, Nepal imports a significant number of small food animals, including sheep and goats, from China to accommodate rising demand during festival seasons, which might increase the risk of zoonotic disease transmission. Therefore, governing authorities must be vigilant on border screening and institute one health approach-based human and animal disease surveillance. A molecular-based assay must be accessible to diagnose such a possible infection in Nepal. Further, large-scale research is critically needed to investigate and comprehend the various components of this virus implicated in human illness, including the severity of infection and mechanism of transmission.

Globally, it is estimated that 70% of emerging infectious illnesses are transmitted to humans through interactions with animals as human populations expand into natural areas [17]. According to the CDC, more than six out of every ten recognized infectious diseases in humans can be spread from animals. Three of every four new or emerging infectious diseases impacting humans are zoonotic or of zoonotic origin [18]. The catastrophic impacts of these infections highlight the necessity of strengthening one health approach-based disease surveillance system. Early identification of outbreaks caused by new viruses and instituting the risk communication mechanisms will be key in preventing them from turning into pandemics [19].

To put things in perspective, amidst the two major viral outbreaks plaguing the world at present time, the emergence of a new virus belonging to a genus that houses the Nipah virus, one of the most fatal infections of all time, can be a cause of concern. Despite the possibility of severe manifestations in immunocompromised, it must be noted here that based on the current knowledge, LayV is not fatal. Although current evidence suggests that LayV is not transmitted from human-to-human, there is a possibility that the virus may evolve and acquire this trait. Nipah, for example, was not transmitted from human-to-human in the first outbreak described from Malaysia, but a high percentage of human-to-human transmission was described in India and Bangladesh in the latter outbreaks [20]. Therefore, there is a need for careful monitoring and surveillance, especially in China and neighboring countries, to understand the evolution of this outbreak. There is also a need for the development of appropriate diagnostics and research into possible treatment options for this disease. The clinical presentations overlap with COVID-19 but not with monkeypox. In areas with high transmission of COVID-19, it is easy to miss this infection. It is, therefore, important to test patients in China and neighbouring regions with a history of animal contact who present with fever and respiratory manifestations but are negative for COVID-19.
